# Neutrophil-to-Lymphocyte Ratio (NLR) and Platelet-to-Lymphocyte Ratio (PLR) as Prognostic Markers of COVID-19 Disease Irrespective of Immunosuppression Status: A Case-Control Retrospective Single-Center Study

**DOI:** 10.3390/pathogens14060550

**Published:** 2025-06-01

**Authors:** Amalia Papanikolopoulou, Vasiliki Rapti, Polyxeni Alexiou, Charalampos M. Charalampous, Maria Effrosyni Livanou, Vissaria Sakka, Konstantinos N. Syrigos, Garyfallia Poulakou

**Affiliations:** Third Department of Internal Medicine and Laboratory, School of Medicine, Sotiria General Hospital, National and Kapodistrian University of Athens, 11527 Athens, Greece; amaliapapaniko@yahoo.com (A.P.); xenialexiou@hotmail.com (P.A.); chacharalampous@gmail.com (C.M.C.); mirsinilivanou20@gmail.com (M.E.L.); vissaria@gmail.com (V.S.); ksyrigos@med.uoa.gr (K.N.S.); gpoulakou@gmail.com (G.P.)

**Keywords:** SARS-CoV-2, COVID-19, neutrophil-to-lymphocyte ratio (NLR), platelet-to-lymphocyte ratio (PLR), prognostic marker, intubation, death, immunosuppression

## Abstract

Neutrophil-to-lymphocyte ratio (NLR) and platelet-to-lymphocyte ratio (PLR) have been studied as predictors for severe COVID-19 outcomes. The aim of the present study is to identify prognostic cut-off values of NLR and PLR for intubation and death in hospitalized COVID-19 patients, with or without immunosuppression. From June 2021 to December 2022, we retrospectively analyzed 393 consecutively admitted COVID-19 patients, who were divided in two cohorts according to immunosuppression status (hematological malignancy and/or autoimmune condition vs. non-immunocompromised), using a propensity score-matching in 1:2 ratio. Higher NLR and PLR values were observed on days 1 and 4 for severe COVID-19, irrespective of immunosuppression status. NLR on day 1 >5.06 and day 4 >6.40 (*p* < 0.001), as well as PLR on day 1 >262.2 and day 4 >217.3 (*p* = 0.003), were associated with a greater probability for intubation. Similarly, a higher probability for death was found in the subset of patients with NLR on day 1 >4.82 (*p* < 0.001) and day 4 >6.41 (*p* < 0.001) and PLR on day 1 >229 (*p* = 0.009) and day 4 >205.4 (*p* = 0.003). Both PLR and NLR exhibited consistently higher negative predictive values (NPVs) (>93%) compared to positive predictive values (PPVs) for intubation and death. NLR and PLR displayed strong prognostic potential in hospitalized COVID-19 patients regarding intubation and death, irrespective of immunosuppression status, thus the surveillance of these biomarkers may help clinicians identify high-risk COVID-19 patients at an early stage.

## 1. Introduction

The coronavirus disease 2019 (COVID-19) pandemic is a global health crisis that continues to spread and significantly impact countries around the world. It is caused by a novel coronavirus, severe acute respiratory syndrome coronavirus 2 (SARS-CoV-2), which was first identified in Wuhan, China, in December 2019 [1]. The clinical course of COVID-19 can range from mild upper respiratory tract infection to a hyperinflammatory response, often referred to as a cytokine storm, with severe respiratory failure and multi-organ dysfunction [2]. Elevated levels of cytokines, such as interleukin 6 (IL-6) and tumor necrosis factor alpha (TNF-a) suppress neutrophil apoptosis, reduce lymphocyte survival and can stimulate platelet production [3,4]. Thus, the exacerbation of severe SARS-CoV-2 infection is characterized by various complete blood count alterations with dynamic phases [5,6].

NLR is a simple biomarker which combines two faces of the immune system: the innate immune response, due to neutrophils, and the adaptive immunity, due to lymphocytes [7]. This marker has been found elevated in many conditions characterized by tissue damage that activates systemic inflammatory response syndrome (SIRS), like bacterial or fungal infection, acute stroke, myocardial infarction, atherosclerosis, severe trauma and cancer [8]. In SARS-CoV-2 infection, NLR has emerged as a significant prognostic marker in assessing the severity and outcomes of the disease [9]. Several studies and a recent meta-analysis involving over 12,000 patients have demonstrated that elevated NLR values correlate with worse clinical outcomes, including higher mortality rates [10,11,12].

PLR is another easily accessible biomarker from the peripheral blood reflecting the balance between systemic inflammation and immunity. It has been linked with the hyperinflammatory state of rheumatologic diseases and found to be predictive of mortality in patients with cancer [13,14]. It has also been used as an indicator of the severity of COVID-19 and associated with worse prognoses [6,15].

On the other hand, the evolving nature of the pandemic, with varying infection rates and the prevalence of severe cases over time, has impacted the ability to generalize the findings [16]. The optimal cut-off values of these markers for predicting COVID-19 outcomes vary across studies, reflecting differences in patient populations and methodologies [16,17]. Also, many confounders which drive these biomarkers upwards have been described, like age, exogenous steroid intake, endogenous sexual hormones, active hematological disorders, human immunodeficiency virus (HIV), acute myocardial infarction, stroke, pulmonary embolism, type 2 diabetes and cancer [18,19].

The aim of the present study was to assess hematological parameters and optimal cut-off values as prognostic tools in COVID-19, comparing two cohorts of hospitalized patients with or without immunosuppression, after the implementation of mass-vaccination programs against SARS-CoV-2 in Greece.

## 2. Materials and Methods

### 2.1. Study Setting

This study retrospectively analyzed patients diagnosed with COVID-19 who were sequentially admitted to the Infectious Diseases Department of a reference General Hospital in Athens, Greece, from June 2021 to December 2022. During this period, the displacement of the Delta by the Omicron variant occurred, and the implementation of mass-vaccination programs against COVID-19 was recorded.

### 2.2. Study Population and Data Collection

We have collected 400 adult patients aged 18 years and above who had confirmed SARS-CoV-2 infection and were hospitalized during the third and fourth wave of the pandemic, characterized by the predominance of the Delta and Omicron variant, respectively. Exclusion criteria were (i) being younger than 17 years of age, (ii) no laboratory-confirmed COVID-19, and (iii) hospitalization during the first or second pandemic wave. We retrospectively gathered the following data for each COVID-19 case from routine patient charts: demographics, concomitant diseases, including the prior medical history of either hematological malignancy (HM) or autoimmune condition (AC), and relative treatment, COVID-19 infection symptoms and outcomes, COVID-19 vaccination status (including the number of doses received and the date of the last dose administered prior to admission), and biochemical and hematological laboratory findings. The following biomarkers have been identified: hematological (lymphocyte count, neutrophil count, NLR, PLR, inflammatory (C-reactive protein [CRP], procalcitonin [PCT]), biochemical (d-dimer, troponin, aspartate aminotransferase [AST], alanine transaminase [ALT], serum creatinine), and those related to coagulation cascades in disseminated intravascular coagulation (DIC).

### 2.3. Definitions

Upon admission, the severity of COVID-19 was evaluated based on clinical indicators and the patients’ oxygen needs. Severe disease was characterized by patients meeting one or more of the following criteria: (i) oxygen saturation (SpO2) ≤ 94% on room air as measured by pulse oximetry, (ii) a ratio of arterial oxygen partial pressure (PaO2) to fractional-inspired oxygen (FiO2) < 300 mm Hg, (iii) tachypnea (respiratory rate ≥ 30 breaths per minute), or (iv) lung infiltrates exceeding 50%. Moderate disease was determined by evidence of lower respiratory tract involvement during clinical assessment or imaging, with SpO2 ≥ 94% on room air [20].

Full vaccination was characterized by the receipt of a primary vaccination series consisting of two doses of BNT162b2 (Comirnaty, Pfizer-BioNTech, New York, NY, USA), mRNA-1273 (Spikevax, Moderna, Cambridge, MA, USA), or ChAdOx1-S (Vaxzevria, AstraZeneca, Cambridge, UK), followed by a booster dose administered at least six months later (from October 2021 onwards). Patients classified as “boosted vaccinated” were those who had received three or more vaccine doses.

Elevations greater than three times the upper limit of normal for ALT or AST were identified as indicative of significant liver dysfunction [21].

Acute kidney injury was defined as an increase in serum creatinine by ≥0.3 mg/dL or ≥1.5 times baseline within 48 h or a urine volume <0.5 mL/kg/h for 6 h [22].

In this study, the classification of SARS-CoV-2 variants (Delta or Omicron) among hospitalized patients was based on the predominant circulating strains in Greece during the corresponding periods of admission, as reported by national epidemiological surveillance data. Specifically, patients admitted during the time frame when the Delta variant was the dominant strain were categorized accordingly, while those hospitalized during the period of Omicron dominance were classified as Omicron cases. It is important to note that no molecular confirmation (e.g., whole genome sequencing or variant-specific PCR) was performed to definitively identify the infecting variant in individual patients.

### 2.4. Outcomes

We sought to identify effective hematological parameters as prognostic tools in COVID-19 and validate cut-off values, investigating possible correlation with the immunodeficient status of the patients, along with the risk for COVID-19 progression, intubation and death.

### 2.5. Statistical Analysis

Quantitative variables were expressed as mean values (Standard Deviation, SD) and as median (interquartile range, IQR), while categorical variables were expressed as absolute and relative frequencies. Propensity score-matching in a 1:2 ratio according to the nearest neighbor matching method was used to match immunocompromised with non-immunocompromised patients. The propensity score was estimated using logistic regression with being immunocompromised (yes vs. no) as the outcome and gender and age as independent variables. For the comparison of proportions, chi-square and Fisher’s exact tests were used. Student’s *t*-tests and Mann–Whitney tests were used for the comparison of continuous variables between two groups. Receiver operating characteristic (ROC) curves were used in order to estimate the prognostic ability of NLR and PLR values on days 1 and 4 for intubation and death. Sensitivity, specificity, positive prognostic and negative prognostic values were calculated for optimal cut-offs. The area under the curve (AUC) was also calculated. Life table analyses were used to calculate cumulative recovery rate (standard errors, SE) for specific time intervals. Kaplan–Meier survival estimates for survival were graphed over the follow-up period. Logistic regression analysis in a stepwise method (*p* for entry 0.05, *p* for removal 0.10) was used in order to find independent factors associated with intubation and death. Adjusted odds ratios (aOR) with 95% confidence intervals (95% CI) were computed from the results of the logistic regression analyses. All reported *p* values are two-tailed. Statistical significance was set at *p* < 0.05, and analyses were conducted using SPSS statistical software (version 26.0).

### 2.6. Ethical Issues

The study protocol received approval from the Institutional Review Board of SOTIRIA General Hospital (approval number: 16982/06-06-2024) and adhered to the principles outlined in the Helsinki Declaration of Human Rights. Due to the retrospective nature of the study and the anonymous analysis of clinical data, the requirement for informed consent forms was waived.

## 3. Results

**Figure 1:**
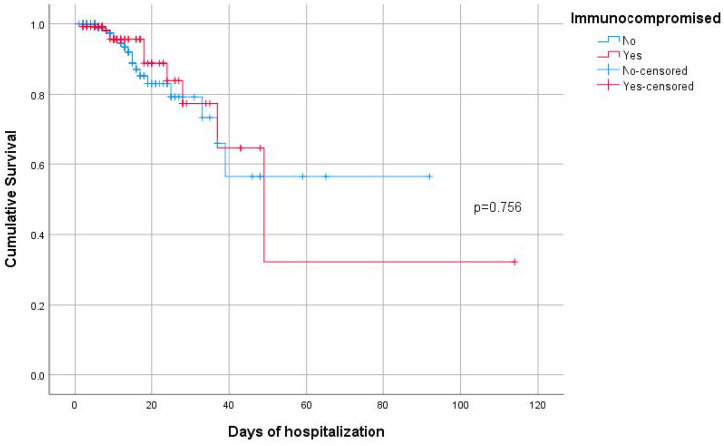
Kaplan–Meier survival curves for immunocompromised and non-immunocompromised patients.

In total, 393 of 400 collected patients (immunocompromised with HM or AC: n = 131, 33.3%; non-immunocompromised: n = 262, 66.7%) were analyzed. Their characteristics are depicted in Table 1. The majority of the patients were males n = 199 (50.6%), and the mean age was 64.7 years (SD = 16.1). According to the World Health Organization (WHO) criteria [20], most of the patients (n = 211, 54%) had severe COVID-19 and were vaccinated against COVID-19 (n = 209, 53.9%). The percentage of any vaccination against COVID-19, or fully (3 doses), or boosted (>3 doses) was significantly greater in the immunocompromised patients, while the group of non-immunocompromised patients were more likely to be partially vaccinated (≤2 doses). All patients received remdesivir for COVID-19, with significant greater duration of treatment in the immunocompromised group according to physician choice, due to the underlying disease and the longer duration on steroids therapy. On the other hand, tocilizumab was an add-on therapy against COVID-19, mostly for non-immunocompromised patients. Concomitant diseases were more significantly encountered in immunocompromised patients, which also experienced longer duration of hospitalization and had greater probability of a secondary bacterial infection. Overall, 44 (11.5%) patients were intubated and 30 patients (7.6%) died, without significant difference between the two groups (*p* = 0.972 and 0.999 respectively). Also, mean hospitalization time till death for non-immunocompromised patients was 63.6 days (SE = 7.1), and for immunoconficompromised patients it was 61.9 (SE = 16.4), without being significantly different from each other (*p* = 0.756) (Figure 1).

Hematological and biochemical parameters in total sample and by group are presented in Table 2. Comparing NLR and PLR values on days 1 and 4 of hospitalization were not significantly different between the two groups. In contrast, higher NLR values on day 1 were observed for Omicron variant and higher NLR and PLR values on days 1 and 4 were observed for patients with severe COVID-19 (Table 3). The percentage of having d-dimers > 1 on day 1 was significantly greater in the immunocompromised group, while the percentage of having fibrinogen > 400 on day 4 was significantly lower in the aforementioned group. Furthermore, the percentages of liver dysfunction and renal dysfunction were significantly lower in the immunocompromised group, while thrombocytopenia was significantly higher (Table 2).

**Figure 2:**
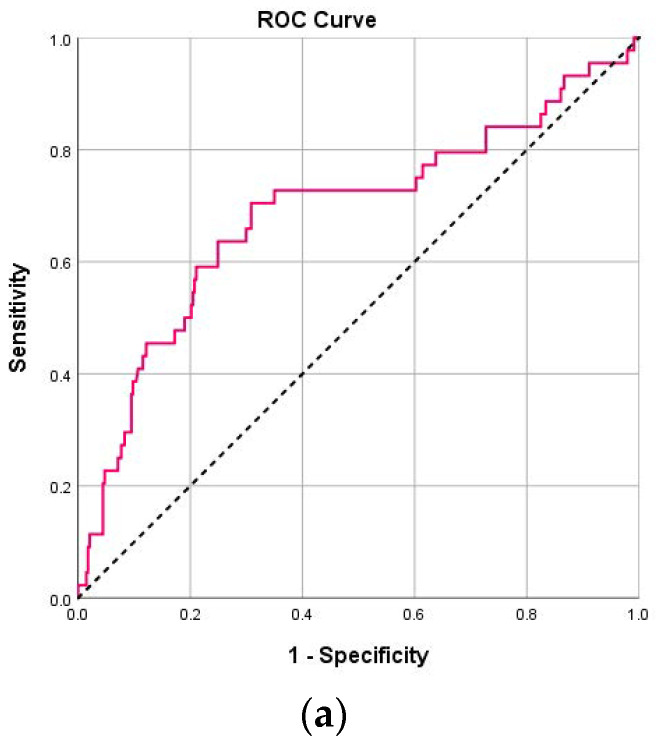

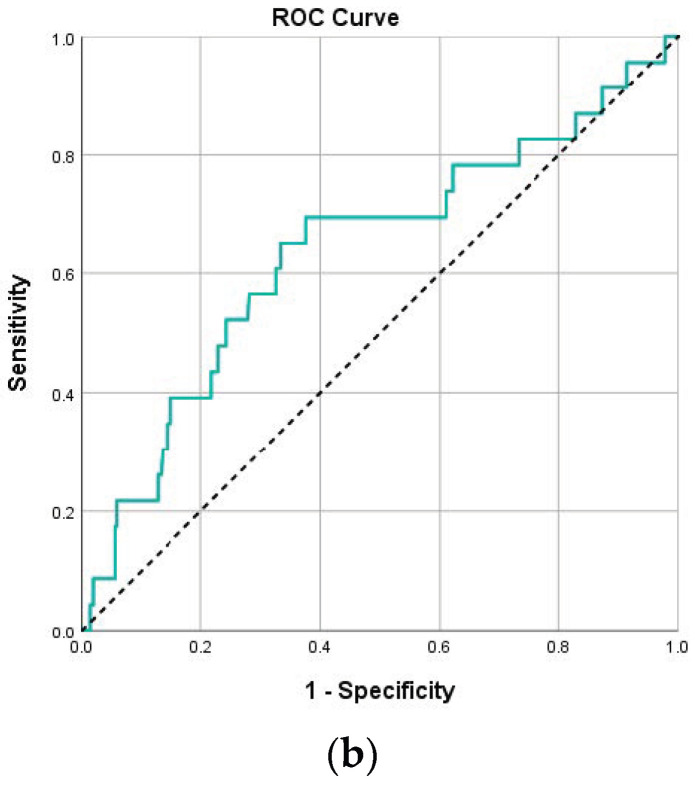
(**a**) ROC curves for NLR values on day 1 for intubation. (**b**) ROC curves for NLR values on day 1 for death.

Regarding intubation, both NLR and PLR values (on both days) had significant prognostic ability (Table 4). More specifically, for NLR on day 1, optimal cut-off was 5.06, with sensitivity 70.5%, specificity 68.8%, PPV 22.8% and NPV 94.7% (Figure 2a). For NLR on day 4, optimal cut-off was 6.40, with sensitivity 60%, specificity 86.7%, PPV 34.4% and NPV 94.9%. Patients with NLR on day 1 greater than 5.06 had 5.27 times greater probability for intubation (95% CI: 2.65–10.48; *p* < 0.001), and patients with NLR on day 4 greater than 6.40 had 9.79 times greater probability for intubation (95% CI: 4.61–20.80; *p* < 0.001). For PLR on day 1, optimal cut-off was 262.2, with sensitivity 59.1%, specificity 76.9%, PPV 25% and NPV 93.5%. For PLR on day 4, optimal cut-off was 217.3, with sensitivity 61.1%, specificity 72.1%, PPV 20.8% and NPV 93.9%. Patients with PLR on day 1 greater than 262.2 had 4.80 times greater probability for intubation (95% CI: 2.50–9.21; *p* < 0.001), and patients with PLR on day 4 greater than 217.3 had 4.06 times greater probability for intubation (95% CI: 1.98–8.31; *p* < 0.001).

**Figure 3:**
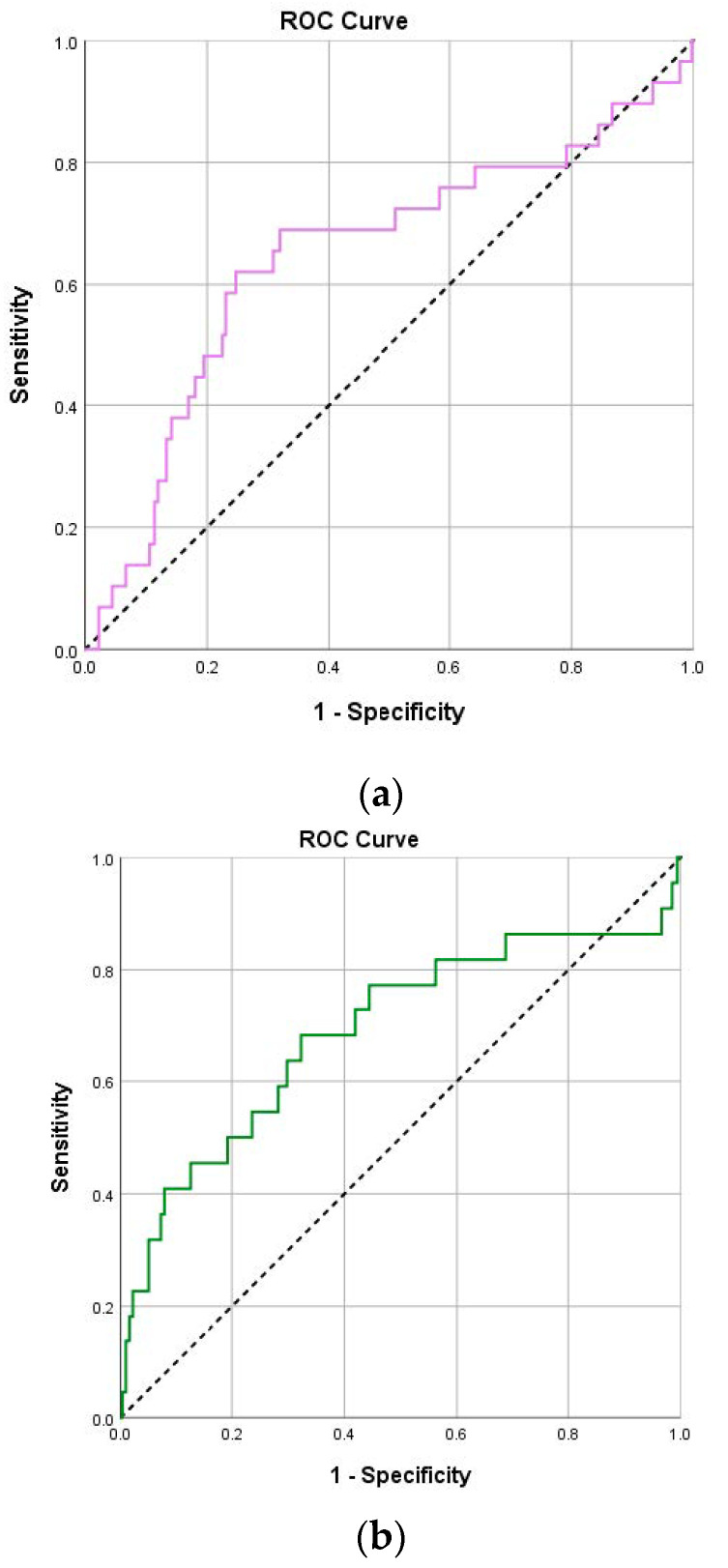
(**a**) ROC curves for PLR values on day 1 for death. (**b**) ROC curves for PLR values on day 4 for death.

Also, regarding death, both NLR and PLR values (on both days) had significant prognostic ability (Table 5). More specifically, for NLR on day 1, optimal cut-off was 4.82, with sensitivity 75.9%, specificity 65%, PPV 14.9% and NPV 97.1% (Figure 2b). For NLR on day 4, optimal cut-off was 6.41, with sensitivity 68.2%, specificity 84.1%, PPV 22.7% and NPV 97.5%. For PLR on day 1, optimal cut-off was 229, with sensitivity 69%, specificity 68.1%, PPV 14.8% and NPV 96.5% (Figure 3a). For PLR on day 4, optimal cut-off was 205.4, with sensitivity 68.2%, specificity 67.7%, PPV 12.6% and NPV 96.9% (Figure 3b). Patients with NLR on day 1 greater than 4.82 had 5.84 times greater probability to die (95% CI: 2.43–14.04; *p* < 0.001), and patients with NLR on day 4 greater than 6.41 had 11.35 times greater probability to die (95% CI: 4.41–29.21; *p* < 0.001). Patients with PLR on day 1 greater than 229 had 4.73 times greater probability to die (95% CI: 2.09–10.72; *p* < 0.001), and patients with PLR on day 4 greater than 205.4 had 4.49 times greater probability to die (95% CI: 1.78–11.35; *p* = 0.001).

Multiple logistic regression revealed that COVID-19 severity and NLR and PLR values on day 1 were significantly associated with intubation (Table 6). Patients with severe COVID-19 had 6.15 times greater probability for intubation (95%CI: 2.31–16.34; *p* < 0.001), patients with NLR on day 1 greater than 5.06 had 2.86 times greater probability (95%CI: 1.27–6.44; *p* = 0.011) and patients with PLR on day 1 greater than 262.2 had 2.22 times greater probability (95%CI: 1.02–4.85; *p* = 0.044) for intubation. Also, multiple logistic regression showed that age, smoking, COVID-19 severity, taking anti-CD20 monotherapy and CRP and NLR values on day 1 were significantly associated with death. There was a greater probability of dying in patients with severe COVID-19 (OR = 5.65; 95%CI: 1.17–27.25; *p* = 0.031), older patients (OR = 1.08; 95%CI: 1.04–1.13; *p* < 0.001), patients who were currently smokers (OR = 3.88; 95%CI: 1.01–16.85; *p* = 0.049), patients who took anti-CD20 monotherapy (OR = 220.28; 95%CI: 13.07–3713.87; *p* < 0.001), patients with CRP on day 1 more than 10 (OR = 2.61; 95%CI: 1.01–6.78; *p* = 0.049) and patients with NLR on day 1 more than 4.82 (OR = 3.92; 95%CI: 1.37–11.2; *p* = 0.011). On the contrary, patients who were vaccinated against COVID-19 had a significantly lower probability of dying by 67% (OR = 0.33; 95%CI: 0.12–0.90; *p* = 0.031). Interestingly, being immunocompromised was not significantly associated either with intubation (OR = 0.74; 95%CI: 0.36–1.50; *p* = 0.400) or death (OR = 0.72; 95%CI: 0.27–1.92; *p* = 0.510).

## 4. Discussion

Our study was conducted in a reference COVID-19 Infectious Diseases Department of a tertiary General Hospital in Athens, Greece, from June 2021 to December 2022. Comparing two cohorts of patients, immunocompromised vs. non- immunocompromised, we aimed to establish the prognostic value of NLR and PLR in COVID-19 severity and validate NLR and PLR cut-off values for the primary outcomes of intubation and death. Despite the difference in immunosuppression status, NLR and PLR performed consistently on days 1 and 4 of hospitalization between the two groups. Patients with immunosuppression were more prone to concomitant disease, secondary infections and thrombocytopenia, while immunocompetent patients had greater liver and renal dysfunction. On the other hand, these biomarkers varied significantly by COVID-19 severity, indicating their strong potential as prognostic markers in COVID-19. Same results are mentioned in the study of Giacaman et al., where significant increment (*p* value ≤ 0.001) on the median of NLR and PLR in immunosuppressed and immunocompetent patients with COVID-19 was observed [23]. Lymphopenia, thrombocytopenia, and NLR were indicative of COVID-19 irrespective of immunosuppression status, while the reduction of lymphocytes and platelet count was more severe in immunosuppressed patients with COVID-19 [23]. It would be expected that the diagnostic and prognostic accuracy of NLR and PLR could be reduced in immunosuppressed COVID-19 patients compared to immunocompetent patients [24]. Other factors, such as the underlying cause of immunosuppression and specific medications used, could further complicate the interpretation of these ratios [25]. In our study, the underlying cause of immunosuppression did not seem to affect either the values of NLR and PLR or the outcomes of intubation and death. Only the anti-CD20 treatment had the greater probability for death.

The optimal cut-off values of NLR and PLR for predicting COVID-19 outcomes vary across studies, reflecting differences in patient demographics, like ethnicity, and in clinical settings, like variations in the definition of disease severity [12,26]. Also, special COVID-19 populations with cardiovascular diseases and risk factors or pre-existing comorbidities may not reflect the general population [17,27]. For NLR values, thresholds for predicting severity and mortality range from 1.65 to 9.47 [10,16]. In a study involving hospitalized COVID-19 patients, an NLR cut-off of 5.94 was identified as a predictive of high in-hospital mortality, with a sensitivity of 62% and specificity of 64% in the derivation cohort [28]. Another study determined an optimal cut-off of 6.82 for predicting poor outcomes, demonstrating good sensitivity and specificity for differentiating between favorable and unfavorable outcomes in COVID-19 patients [29]. In an African COVID-19 population, it was reported that the highest NLR cut-off of 9.47 was optimal for predicting mortality, with a high sensitivity of 88.7% and specificity [10]. Interestingly, in the study of Colaneri et al., sex-specific cut-offs were estimated, with values ranging from approximately 6.36 to 7.29 for women and men, respectively, indicating the need for tailored approaches in clinical settings [15]. In our study, where the 1/3 of the population were immunocompromised, the estimated NLR cut-off values for COVID-19 outcomes were in accordance with other mentioned studies [28,29]. Peaking two timing measurements, we captured an increasing trend between days 1 and 4 of hospitalization for NLR cut-off values, from 5.06 to 6.40 for intubation, and from 4.82 to 6.41 for death.

Regarding PLR, it has also been associated with disease severity, and higher values are indicative in critically ill or deceased patients compared to survivors and those with mild, longer hospitalization to 274 for severe pneumonia [30,31]. While in the recent study of Colaneri illness [26], diversity in cut-off values characterizes small retrospective studies from China, ranging from 126.7 for et al. Distinct PLR cut-off values were used for men (248.00, 250.39) and women (246.45, 241.54) regarding intubation and death, respectively [15]. In our study, the highest estimated PLR cut-off value was seen in day 1 for intubation (262.2) with a decreasing trend afterwards (day 4: 217.3). Smaller PLR cut-offs were recorded for death, yet performed similarly with a decreasing trend between days 1 and 4 of hospitalization (229, 205.4, respectively). This may reflect the statistically significant greater percentage of thrombocytopenia in the immunocompromised group during hospitalization.

The sensitivity and specificity of NLR and PLR in predicting COVID-19 severity have also been evaluated in several studies. From an early review and meta-analysis, the following pooled values were reported for NLR: sensitivity: 78% (95% CI: 70–84%), specificity: 78% (95% CI: 73–83%) [32]. These values indicate that NLR is a reliable biomarker for identifying patients at risk of severe COVID-19 outcomes. Additionally, the area under the curve (AUC) for NLR in predicting disease severity was found to be 0.85 (95% CI 0.81–0.88), suggesting high diagnostic accuracy [33]. The same results are mentioned in optimal cut-off values for predicting mortality, with a sensitivity of 88.7% and a specificity of 95.4% (AUC: 0.95, 95% CI 0.92–98) [10]. Overall, NLR serves as a valuable tool for clinicians to assess the risk of severe disease in COVID-19 patients, with consistent sensitivity and specificity across various studies. In our study, the sensitivity for NLR was higher on day 1, reaching 70.5% (AUC: 0.69, 95% CI 0.59–0.78) for intubation and 75.9% (AUC: 0.70, 95% CI 0.59–0.81) for death, dropping afterwards on day 4 to 60.0% (AUC: 0.74, 95% CI 0.64–0.85) and 68.2% (AUC: 0.73, 95% CI 0.59–0.87), respectively. On the other hand, specificity for NLR performed reversely and was higher on day 4 both for intubation (from 68.8% day 1 to 86.7%) and death (from 65% day 1 to 84.1%). These results indicate that in a population with partially immunocompromised patients, NLR continues to be a reliable biomarker for identifying patients at risk of severe COVID-19 outcomes.

Regarding PLR, its predictive accuracy is generally lower than that of NLR. While elevated PLR values correlate with worse outcomes, they do not consistently demonstrate the same level of specificity and sensitivity in predicting severe COVID-19 cases [6,15]. In our study, only the sensitivity for intubation on day 1 and 4 was near 60%, while specificity was reaching 76.9% and 72.1%, respectively. For the outcome of death, the percentages of sensitivity (69% to 68.2%) and specificity (68.1% to 67.7%) were very similar, showing efficacy in predicting worse outcomes.

Additionally, both for PLR and NLR, PPVs generally remained low for both outcomes and both time points, while NPVs were ranging in high levels from 93.5% to 97.5%. The high NPV underscores the efficacy of PLR and NLR in identifying patients less prone to developing severe COVID-19 irrespective of immunocompromised status, emphasizing their valuable role in risk stratification and clinical decision-making [15].

Another important issue is the timing of NLR measurement and how it impacts the predictive value of NLR and PLR in COVID-19. Some studies showed that initial measurements taken at hospital admission have more prognostic value in COVID-19 patients when compared to Influenza and RSV, with a potential to rise over time [28]. The timing of hospitalization relative to symptom onset can impact NLR measurements. Patients admitted earlier in the disease course may have lower baseline NLR values compared to those admitted later [16]. Also, treatments administered before hospitalization, such as steroids, may influence peripheral blood biomarkers like NLR, potentially leading to underestimation or overestimation of its predictive value [18,32]. In contrast, follow-up NLR measurements, taken later in the disease course, have shown stronger predictive capabilities for in-hospital mortality compared to baseline values [17]. An increasing NLR trend over successive timepoints may reflect disease severity or mortality risk [6]. In our study, NLR cut-offs investigated for intubation and death performed similarly, with an increasing trend and higher AUCs during hospitalization. Also, PLR cut-offs for both outcomes performed similarly, but with a decreasing trend, without affecting the diagnostic accuracy of AUCs, which were again higher on day 4 of hospitalization. This highlights that in severe cases, higher NLR and PLR cut-off values or corresponding AUCs at sequential time points could correlate with worsening conditions and higher mortality rates [6]. Finally, from multiple logistic regression analysis, baseline NLR and PLR values confer greater probability for severe COVID-19 outcomes along with other known variables like age, smoking, COVID-19 severity, vaccination status, inflammation markers (e.g., CRP), and anti-CD20 immunosuppression treatment [34].

While this study provides meaningful insights and contributes to the understanding of prognostic hematological markers in COVID-19, several limitations should be acknowledged. First, the retrospective design may introduce inherent biases and limitations in data collection. Second, variability in the management of underlying immunocompromising conditions—as well as the activity status of hematological malignancies or autoimmune diseases at the time of COVID-19 infection—could influence the generalizability of our findings. Third, the absence of external validation from other geographical regions limits the broader applicability of the proposed cut-off values. Lastly, the classification of SARS-CoV-2 variants was based on national epidemiological trends rather than molecular confirmation, which may have introduced potential misclassification bias. Ongoing research is directed toward addressing these limitations and further evaluating the clinical utility of NLR and PLR in the risk stratification and management of COVID-19.

## 5. Conclusions

Hematological findings and complications of COVID-19 are documented early in the disease. NLR and PLR stand out among various biomarkers for their simplicity, cost-effectiveness, and strong potential as prognostic markers in COVID-19. This correlation with COVID-19 severity and mortality is irrespective of immunosuppression status of the patients, which could act as a false positive confounder. Surveillance of these biomarkers may help clinicians identify high-risk COVID-19 patients at an early stage. The high NPVs of NLR and PLR underscore their efficacy in identifying patients less prone to developing severe COVID-19, emphasizing their valuable role in clinical decision-making. Additional research is needed to establish specific cut-off values and interpret NLR and PLR across diverse populations with COVID-19.

## Figures and Tables

**Table 1:** Study population characteristics classified by immunosuppression status.

	Total Sample n = 393	Immunocompromised		
		No (n = 262; 66.7%)	Yes (n = 131; 33.3%)	
	n (%)	n (%)	n (%)	*p*-Value
Gender				
Male	199 (50.6)	139 (53.1)	60 (45.8)	0.175 +
Female	194 (49.4)	123 (46.9)	71 (54.2)	
Age (years), mean (SD)	64.7 (16.1)	64.3 (16.3)	65.4 (15.7)	0.544 ‡
Smoking				
No	286 (73.7)	201 (77.3)	85 (66.4)	<0.001 +
Yes	51 (13.1)	41 (15.8)	10 (7.8)	
In the past	51 (13.1)	18 (6.9)	33 (25.8)	
Vaccination status				
Any vaccination against COVID-19	209 (53.9)	124 (48.1)	85 (65.4)	0.001 +
Partially vaccinated (≤2 doses)	63 (16.0)	57 (21.8)	6 (4.6)	<0.001 +
Fully vaccinated (3 doses)	116 (29.5)	57 (21.8)	59 (45.0)	<0.001 +
Boosted vaccinated (>3 doses)	30 (7.6)	10 (3.8)	20 (15.3)	<0.001 +
Duration from last vaccine dose to hospitalization (days), median (IQR)	9 (0–174)	0 (0–171)	69 (0–185)	0.049 ‡
COVID-19 disease				
Mild/Moderate	180 (46)	127 (48.8)	53 (40.5)	0.116 +
Severe	211 (54)	133 (51.2)	78 (59.5)	
Variant				
Delta	141 (35.9)	98 (37.4)	43 (32.8)	0.372 +
Omicron	252 (64.1)	164 (62.6)	88 (67.2)	
Anti-CD 20 monotherapy	5 (1.3)	0 (0)	5 (3.8)	0.004 ++
COVID-19 treatment				
Duration of Remdesivir (days), median (IQR)	5 (5–5)	5 (5–5)	5 (5–10)	<0.001 ‡
Tocilizumab	54 (13.7)	46 (17.5)	8 (6.1)	0.002 +
Baricitinib	23 (5.9)	18 (6.9)	5 (3.8)	0.221 +
Anakinra	25 (6.4)	15 (5.7)	10 (7.6)	0.471 +
Steroids	173 (44.0)	123 (47.0)	50 (38.2)	0.063 +
Long duration on steroid therapy	49 (12.5)	20 (7.6)	29 (22.1)	<0.001 +
Concomitant disease	306 (77.9)	175 (66.8)	131 (100)	<0.001 +
Secondary infections	94	0	94	<0.001 ++
Baseline WHO stage				
3	177 (45.0)	124 (47.3)	53 (40.5)	0.581 ++
4	196 (49.9)	124 (47.3)	72 (54.9)	
5	17 (4.3)	12 (4.6)	5 (3.8)	
6	3 (0.7)	2 (0.7)	1 (0.8)	
Maximum oxygen therapy				
No	117 (29.8)	69 (26.3)	48 (36.6)	0.091 ++
Nasal cannula	128 (32.6)	88 (33.6)	40 (30.5)	
Venturi Mask	65 (16.5)	46 (17.6)	19 (14.5)	
HFNC	30 (7.6)	25 (9.5)	5 (3.8)	
Non-rebreather mask	5 (1.2)	2 (0.8)	3 (2.3)	
Intubation	44 (11.2)	29 (11.7)	15 (11.5)	
Discharge oxygen therapy				
No oxygen need	324 (82.4)	214 (81.7)	110 (84.0)	0.013 ++
Oxygen supply	36 (9.2)	20 (7.6)	16 (12.2)	
Duration of hospitalization (days), median (IQR)	9 (6–14)	8 (5–14)	10 (7–16)	0.036 ‡‡
Duration of intubation (days), median (IQR)	12 (6–29)	12 (7–31)	10 (3–23)	0.386 ‡‡
Intubation	44 (11.2)	29 (11.7)	15 (11.5)	0.972
Death	30 (7.6)	20 (7.6)	10 (7.6)	>0.999 +

COVID-19: coronavirus disease 2019; HFNC: high-flow nasal cannula; IQR: interquartile range; SD: standard deviation; WHO: World Health Organization. + Pearson’s chi-square test; ++ Fisher’s exact test; ‡ Student’s *t*-test; ‡‡ Mann–Whitney test.

**Table 2:** Hematological and biochemical parameters in the study population classified.

	Total Sample n = 393	Immunocompromised		
		No (n = 262; 66.7%)	Yes (n = 131; 33.3%)	
	n (%)	n (%)	n (%)	*p*-Value
NLR (day 1), median (IQR)	3.83 (2.24–6.89)	3.7 (2.34–6.48)	4.04 (1.86–8.53)	0.812 ‡‡
NLR (day 4), median (IQR)	2.5 (1.5–5.5)	2.62 (1.62–5.75)	2.3 (1.41–5.24)	0.275 ‡‡
PLR (day 1), median (IQR)	175.3 (114.7–269.3)	173.5 (114.7–256.5)	180 (114.6–296.6)	0.464 ‡‡
PLR (day 4), median (IQR)	153.9 (97.7–245.2)	158.5 (103.9–246.7)	148.3 (83.4–244)	0.284 ‡‡
D-DIM (day 1)				
<1	236 (68.4)	172 (71.7)	64 (61)	0.049 +
≥1	109 (31.6)	68 (28.3)	41 (39)	
D-DIM (day 4)				
<1	77 (64.2)	53 (63.1)	24 (66.7)	0.708 +
≥1	43 (35.8)	31 (36.9)	12 (33.3)	
D-DIM (day 1)				
<0.5	130 (37.7)	96 (40)	34 (32.4)	0.179 +
>0.5	215 (62.3)	144 (60)	71 (67.6)	
D-DIM (day 4)				
<0.5	48 (40)	35 (41.7)	13 (36.1)	0.569 +
>0.5	72 (60)	49 (58.3)	23 (63.9)	
PCT (day 1)				
<0.25	147 (91.3)	104 (93.7)	43 (86)	0.133 ++
>0.25	14 (8.7)	7 (6.3)	7 (14)	
CRP (day 1)				
≤10	311 (81)	208 (81.9)	103 (79.2)	0.530 +
>10	73 (19)	46 (18.1)	27 (20.8)	
CRP (day 4)				
≤10	293 (87.2)	201 (89.3)	92 (82.9)	0.096 +
>10	43 (12.8)	24 (10.7)	19 (17.1)	
Fibr (day 1)				
≤400	19 (19.0)	10 (14.7)	9 (28.1)	0.111 +
>400	81 (81.0)	58 (85.3)	23 (71.9)	
Fibr (day 4)				
≤400	26 (24.5)	14 (18.9)	12 (37.5)	0.041 +
>400	80 (75.5)	60 (81.1)	20 (62.5)	
Fibr (day 1)				
≤600	71 (71)	45 (66.2)	26 (81.3)	0.121 +
>600	29 (29)	23 (33.8)	6 (18.8)	
Fibr (day 4)				
≤600	87 (82.1)	60 (81.1)	27 (84.4)	0.685 +
>600	19 (17.9)	14 (18.9)	5 (15.6)	
Liver dysfunction	60 (15.5)	48 (18.8)	12 (9.2)	0.014 +
Acute kidney injury	62 (16.1)	50 (19.7)	12 (9.2)	0.008 +
Thrombocytopenia	102 (26.5)	58 (22.6)	44 (34.4)	0.013 +

CRP: C-reactive protein; D-dim: d-dimers; Fibr: fibrogen; IQR: interquartile range; NLR: neutrophil-to-lymphocyte ratio; PCT: procalcitonin. + Pearson’s chi-square test; ++ Fisher’s exact test; ‡‡ Mann–Whitney test.

**Table 3:** NLR and PLR values by COVID-19 severity and variant.

	Median (IQR)	Median (IQR)	*p*-Value
Variant:	Delta	Omicron	
NLR (day 1)	3.08 (1.95–5.59)	4.14 (2.47–7.95)	0.003
NLR (day 4)	2.43 (1.51–4.94)	2.52 (1.58–5.77)	0.772
PLR (day 1)	180.0 (120.6–244.8)	173.9(114.0–291.1)	0.648
PLR (day 4)	162.8 (108.8–244.0)	147.8 (92.8–246.3)	0.380
COVID-19 disease:	Mild/Moderate	Severe	
NLR (day 1)	2.99 (2.05–5.01)	4.68 (2.62–9.44)	<0.001
NLR (day 4)	1.84 (1.38–3.25)	3.46 (1.93–6.47)	<0.001
PLR (day 1)	157.8 (111.4–215.1)	207.3 (134.2–314.4)	<0.001
PLR (day 4)	127.4 (91.2–204.3)	174.1 (108.6–306.7)	<0.001

COVID-19: coronavirus disease 2019; IQR: interquartile range; NLR: neutrophil-to-lymphocyte ratio; PLR: platelet-to-lymphocyte ratio.

**Table 4:** ROC analysis results and NLR and PLR values by intubation.

	Intubation		*p*						
	No	Yes							
	Median (IQR)	Median (IQR)		AUC (95% CI)	Optimal Cut-Off	Sensitivity (%)	Specificity (%)	PPV (%)	NPV (%)
NLR (day 1)	3.58 (2.21–6.08)	7.29 (2.90–11.95)	<0.001	0.69 (0.59–0.78)	5.06	70.5	68.8	22.8	94.7
NLR (day 4)	2.28 (1.51–4.59)	6.81 (2.55–12.55)	<0.001	0.74 (0.64–0.85)	6.40	60.0	86.7	34.4	94.9
PLR (day 1)	172.6 (114.6–250.4)	268.4 (149.4–369.6)	0.003	0.64 (0.54 – 0.74)	262.2	59.1	76.9	25.0	93.5
PLR (day 4)	145.7 (95.6–227.0)	241.3 (120.1–485.0)	0.003	0.65 (0.54–0.77)	217.3	61.1	72.1	20.8	93.9

AUC: area under curve; CI: confidence interval; IQR: interquartile range; NLR: neutrophil-to-lymphocyte ratio; PLR: platelet-to-lymphocyte ratio; NPV: negative prognostic value; PPV: positive prognostic value.

**Table 5:** ROC analysis results and NLR and PLR values by death.

	Death		*p*						
	No	Yes							
	Median (IQR)	Median (IQR)		AUC (95% CI)	Optimal Cut-Off	Sensitivity (%)	Specificity (%)	PPV (%)	NPV (%)
NLR (day 1)	3.6 (2.2–6.5)	7.5 (4.8–11.8)	**<0.001**	0.70 (0.59–0.81)	4.82	75.9	65	14.9	97.1
NLR (day 4)	2.4 (1.5–5)	9.3 (2.4–18.9)	**<0.001**	0.73 (0.59–0.87)	6.41	68.2	84.1	22.7	97.5
PLR (day 1)	172.7 (114.1–261.9)	270 (156–367.3)	**0.009**	0.65 (0.53–0.76)	229	69	68.1	14.8	96.5
PLR (day 4)	147.8 (96.8–233.4)	255.3 (165.7–541)	**0.003**	0.69 (0.55–0.83)	205.4	68.2	67.7	12.6	96.9

AUC: area under curve; CI: confidence interval; IQR: interquartile range; NLR: neutrophil-to-lymphocyte ratio; PLR: platelet-to-lymphocyte ratio; NPV: negative prognostic value; PPV: positive prognostic value.

**Table 6:** Multiple logistic regression results, in a stepwise method, with intubation and death as dependent variables.

Dependent Variable	Independent Variable	OR (95% CI) +	*p*-Value
Intubation	COVID-19 disease (Severe vs. Mild/Moderate)	6.15 (2.31–16.34)	<0.001
	Immunocompromised (Yes vs. No)	0.74 (0.36–1.5)	0.400
	NLR (day 1) (>5.06 vs. <5.06)	2.86 (1.27–6.44)	0.011
	PLR (day 1) (>262.2 vs. < 262.2)	2.22 (1.02–4.85)	0.044
Death	COVID-19 disease (Severe vs. Mild/Moderate)	5.65 (1.17–27.25)	0.031
	Age (years)	1.08 (1.04–1.13)	<0.001
	Smoking		
	Yes vs. No	3.88 (1.01–16.85)	0.049
	In the past vs. No	2.96 (0.79–11.15)	0.108
	Vaccination against COVID-19 (Yes vs. No)	0.33 (0.12–0.9)	0.031
	Anti-CD 20 monotherapy (RITUXIMAB) (Yes vs. No)	220.28 (13.07–3713.87)	<0.001
	CRP (day 1) (>10 vs. <10)	2.61 (1.01–6.78)	0.049
	NLR (day 1) (>4.82 vs. <4.82)	3.92 (1.37–11.2)	0.011
	Immunocompromised (Yes vs. No)	0.54 (0.17–1.73)	0.301

COVID-19: coronavirus disease 2019; CRP: C-reactive protein; NLR: neutrophil-to-lymphocyte ratio; OR: odds ratio; PLR: platelet-to-lymphocyte ratio.

## Data Availability

Raw data is not available due to patient confidentiality, but can be provided upon request.
